# Short-Term Results of Switch from Conbercept to Bevacizumab or Ranibizumab in Eyes with Persistent Neovascular Age-Related Macular Degeneration

**DOI:** 10.1155/2020/9340356

**Published:** 2020-09-07

**Authors:** Zongyi Wang, Mengyang Li, Yuou Yao, Jie Hu, Jiyang Tang, Ran Tang, Zhenyu Piao, Jinfeng Qu

**Affiliations:** Department of Ophthalmology, Peking University People's Hospital, Eye Diseases and Optometry Institute, Beijing Key Laboratory of Diagnosis and Therapy of Retinal and Choroid Diseases, College of Optometry, Peking University Health Science Center, Beijing 100044, China

## Abstract

**Purpose:**

To study the short-term anatomical and functional outcomes in patients with neovascular age-related macular degeneration (nAMD) who were previously treated with conbercept and switched to ranibizumab or bevacizumab due to persistent activity.

**Methods:**

This retrospective single-arm study included nAMD patients who were followed up for at least three months after switching from at least 3 monthly intravitreal conbercept injections to bevacizumab or ranibizumab for persistent choroidal neovascularization (CNV) activity. The demographic data, treatments, best-corrected visual acuity (BCVA), central macular thickness (CMT), and the height of pigmented epithelial detachment (PED) before and after switching were recorded and analyzed.

**Results:**

A total of 64 eyes of 64 patients were included with a mean follow-up of 9.6 ± 3.0 months. The average number of injections of conbercept was 3.6 ± 0.8 (range, 3–5) before switching. 18 eyes were switched to bevacizumab, and the other 46 eyes were switched to ranibizumab. After switching, mean BCVA slowly improved from 0.73 ± 0.48 to 0.64 ± 0.41 (*p*=0.0132) at one month after the last intravitreal injection of ranibizumab or bevacizumab during the mean follow-up of 4.4 ± 2.0 months. One month after switching, the mean CMT decreased significantly from 294.9 ± 121.8 *μ*m to 230.9 ± 107.0 *μ*m (*p* < 0.0001) and kept stable during the follow-up. There was a significant reduction of maximum PED height (mPEDH) at the first month after switching (from 384.3 ± 340.3 *μ*m to 287.2 ± 245.2 *μ*m, *p*=0.0018) and kept stable during the follow-up. The mean PED height at foveal center (cPEDH) showed a regression over time after switching (from 169.3 ± 230.6 *μ*m to 130.5 ± 180.2 *μ*m, *p*=0.0227) and also kept stable during the follow-up. The proportion of patients with IRF was slightly increased but not statistically significant before switching. After switching, this proportion decreased significantly from 96.9% to 81.3% at one month after the first intravitreal injection of ranibizumab or bevacizumab (*p*=0.0086). The proportion of patients with SRF did not change significantly before and after switching. The mean decrease of mPEDH and cPEDH at the last follow-up after switching was significantly larger in the IVR subgroup than in the IVB subgroup (*p*=0.023 and 0.010).

**Conclusion:**

Our results indicate that switching from intravitreal conbercept injections to bevacizumab or ranibizumab can lead to significant improvement of CMT, PED, and IRF and slight improvement of BCVA in a short period of time for persistent nAMD patients.

## 1. Introduction

Intravitreal injection of antivascular endothelial growth factor (anti-VEGF) has been proved to be effective in improving the visual prognosis of patients with neovascular age-related macular degeneration (nAMD) and was known as the first-line therapy for nAMD [1–4]. Three anti-VEGF agents are currently used worldwide: ranibizumab, bevacizumab, and aflibercept.

Conbercept (Lumit®, Chengdu Kanghong Biotech Co., Ltd., P. R. China) is another anti-VEGF drug which was developed in China and has been approved for the treatment of nAMD by China Food and Drug Regulatory Administration (CFDA) in 2013 and was recently admitted directly to the phase III clinical trials in the U.S. Food and Drug Administration (FDA). Similar to aflibercept, conbercept is a fusion protein that contains extracellular domain 2 of VEGF receptor 1 and extracellular domains 3 and 4 of VEGF receptor 2 fused to the Fc portion of human immunoglobulin G1. It competitively prevents the binding of VEGF to its receptor and inhibits the downstream pathway activation. It has high affinity to all isoforms of VEGF-A and also binds to placental growth factor (PlGF) and VEGF-B [5, 6]. Many studies have shown that conbercept had good efficacy and safety in treating PCV and AMD. It not only had equivalent effects on visual and anatomic improvement comparing to ranibizumab, but also achieved longer treatment intervals in more patients [7–14].

Researchers have tried to improve outcomes for resistant nAMD patients by changing from one anti-VEGF drug to the other, assuming different molecular structure and biochemical properties will make difference in effect. Most of these switching studies report outcomes for patients switching from recombinant antibody of VEGF-A such as ranibizumab and/or bevacizumab to receptor decoy fusion protein such as aflibercept. Although most of these studies reported statistical reduction of central macular thickness (CMT), there is a wide variation in visual outcomes [15–30]. The limitation such as no control arm and retrospective trials with relatively small sample size makes it is challenging to interpret these data. Some may argue that the benefit could just be attribute of natural change overtime rather than switching [30].

As to conbercept, there have not been any switching studies about it. Our purpose is to study the short-term anatomical and functional outcomes in patients with nAMD who were previously treated with conbercept and switched to ranibizumab or bevacizumab due to persistent activity.

## 2. Methods

This retrospective single-arm study included patients who had been followed up for at least six months at the ophthalmology department of People's Hospital of Peking University with a diagnosis of nAMD between April 2015 and February 2020.

Inclusion criteria were (1) treatment naïve nAMD before receiving the first injection of conbercept with active choroidal neovascularization (CNV) confirmed by fluorescein angiography (FA) and spectral domain optical coherence tomography (SD-OCT); (2) eyes treated with at least 3 monthly injections of 0.5 mg conbercept and then following a pro re nata (PRN) regimen; (3) persistence or increase of subretinal fluid (SRF) or intraretinal fluid (IRF) after at least 3 monthly injections of conbercept; (4) eyes then being switched to intravitreal injections of 1.25 mg bevacizumab or 0.5 mg ranibizumab within 1 month regardless of the number of the injections in PRN regimen; (5) patients being followed up for at least three months after switching.

Exclusion criteria were (1) age younger than 50 years; (2) after retinal photocoagulation or vitrectomy; (3) patients with severe systemic disease; (4) follow-up of less than six months; (5) other conditions associated with CNV such as pathologic myopia or angioid streaks and other ocular conditions; (6) eyes underwent cataract surgery during the follow-up.

Baseline examination and all subsequent follow-up visits included best-corrected visual acuity (BCVA), slit-lamp examination, measurement of the intraocular pressure, a dilated fundus examination, and SD-OCT examination. Fundus color photography, fluorescein angiography, and indocyanine green angiography were performed at baseline and at the physician's discretion during the follow-up. BCVA were recorded in decimal and changed to LogMAR scale for statistic calculation. SD-OCT scans were acquired by RTVue XR Avanti (Optovue, Fremont, CA, USA) or Cirrus HD-OCT 5000 (Carl Zeiss Meditec Inc., Dublin, CA, USA), and followed on the same machine at each visit. Each SD-OCT scan from volume scan program was viewed for the presence or absence of SRF, IRF, and pigmented epithelial detachment (PED). Central macular thickness (CMT) was measured as the distance between ILM and the inner border of the RPE at the very fovea center, manually using the build-in caliper on OCT device. The maximum PED height (mPEDH) was measured as the distance between the inner border of the RPE and Bruch's membrane at the highest site of PED. If PED was present at foveal center, PED height at foveal center (cPEDH) was measured as the distance between the inner border of the RPE and Bruch's membrane at the very center of fovea. All measurement was done by the same investigator.

The medical charts of all patients were reviewed in addition to their demographic data including age, sex, and laterality. The subtypes of nAMD at baseline were classified into four categories according to types of new vessels present: type 1 CNV, type 2 CNV, PCV, and mixed type. All patients were recorded, included persistent, recurrent, or worsening of retinal and subretinal exudation, as well as the occurrence of retinal or subretinal hemorrhage. Measurements of BCVA, CMT, mPEDH, and cPEDH were recorded at baseline visit (T0), 1 month after the first injection of conbercept (T1), 1 month after the 3 injections of conbercept (T2), 1 month after the first injection of ranibizumab or bevacizumab (T3), and 1 month after the last intravitreal injection of ranibizumab or bevacizumab (T4).

Qualitative variables were described in percentages and quantitative variables were described by their mean with their standard deviation. Comparisons of means (BCVA, CMT, and PED height) were performed using the paired *t*-test if the distribution of the variables was not normal. A value of *p* < 0.05 was retained as significant. Statistical analyses were performed using GraphPad prism 8.0 software (GraphPad Software Inc., La Jolla, California, USA).

## 3. Results

The baseline demographic characteristics and CNV type are presented in Table 1. The mean follow-up was 9.7 ± 3.0 months.

Before switching, patients were given 3.6 ± 0.8 injections of conbercept in average. Among them, 33 eyes (51.6%) received three intravitreal injections of conbercept (IVC), and 31 (48.4%) received more than 3 times of IVC. 18 eyes were then switched to bevacizumab, and the other 46 eyes were switched to ranibizumab following PRN regimen. The average number was 2.0 ± 1.1 (range, 1–4) for intravitreal injection of bevacizumab (IVB) and 2.3 ± 0.9 (range, 1–4) for intravitreal injection of ranibizumab (IVR) during the mean follow-up of 4.4 ± 2.0 months. 17 eyes (26.6%) received one IVB or IVR, 22 (34.3%) received two IVB or IVR, 17 (26.6%) received three IVB or IVR, and 8 (12.5%) received four IVB or IVR.

**Figure 1:**
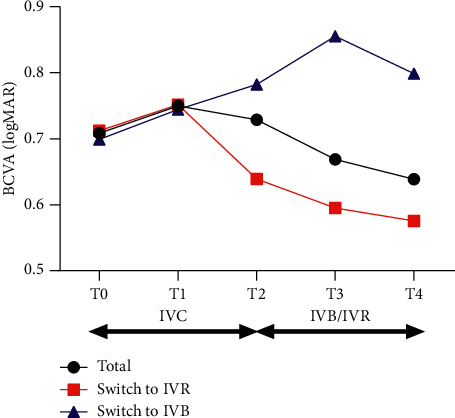
Changes of mean BCVA in eyes with persistent nAMD. There was no significant improvement of BCVA during the treatment of conbercept. After switching to IVR or IVB, BCVA slowly improved from 0.73 ± 0.48 to 0.67 ± 0.41 at T3 (*p*=0.0849) and 0.64 ± 0.41 at T4 (*p*=0.0132). No significant difference was found between IVR and IVB subgroups for the mean changes of BCVA from T2 to T3 (*p*=0.20) and T4 (*p*=0.38). BCVA, best-corrected visual acuity; IVC, intravitreal injections of conbercept; IVB, intravitreal injection of bevacizumab; IVR, intravitreal injection of ranibizumab; T0, baseline visit; T1, 1 month after the first ICV; T2, 1 month after 3 monthly IVC; T3, 1 month after the first IVR or IVB; T4, 1 month after the last IVR or IVB.

For all the patients, the mean BCVA was 0.71 ± 0.38, 0.75 ± 0.58, 0.73 ± 0.48, 0.67 ± 0.41, and 0.64 ± 0.41 at T0, T1, T2, T3, and T4, respectively. There was no significant improvement of BCVA during the treatment of IVC (*p*=0.4389 between T0 and T1, 0.6164 between T0 and T2). After switching, BCVA slowly improved from T2 to T4 (*p*=0.0132) although there was no significant improvement at T3 (*p*=0.0849) (see Figure 1).

For those switched to IVR, mean BCVA increased from 0.64 ± 0.50 at T2 to 0.60 ± 0.42 at T3 and continued increase to 0.58 ± 0.42 at T4. For those switched to IVB, the mean BCVA decreased from 0.78 ± 0.39 at T2 to 0.86 ± 0.31 at T3 but bounced back to 0.80 ± 0.33 at T4. The mean changes of BCVA in IVR and IVB subgroups were −0.04 and 0.07 from T2 to T3 and −0.06 and 0.02 from T2 to T4. No significant difference was found between IVR and IVB subgroups for the mean changes of BCVA from T2 to T3 (*p*=0.20) and T4 (*p*=0.38) (see Figure 1).

**Figure 2:**
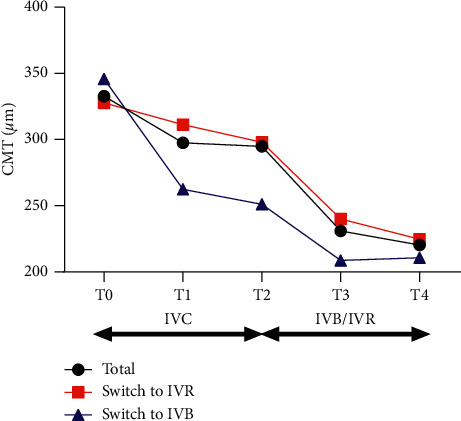
Changes of mean CMT in eyes with persistent nAMD. The mean CMT decreased significantly from T0 to T1 (*p*=0.0149) followed by no further regression. But after switching to IVR or IVB, it decreased significantly from 294.9 ± 121.8 *μ*m at T2 to 230.9 ± 107.0 *μ*m at T3 and 220.4 ± 105.4 *μ*m at T4. No significant difference was found between IVR and IVB subgroups for the mean decrease of CMT from T2 to T3 (*p*=0.53) and T4 (*p*=0.23). CMT, central macular thickness; IVC, intravitreal injections of conbercept; IVB, intravitreal injection of bevacizumab; IVR, intravitreal injection of ranibizumab; T0, baseline visit; T1, 1 month after the first ICV; T2, 1 month after 3 monthly IVC; T3, 1 month after the first IVR or IVB; T4, 1 month after the last IVR or IVB.

For all the patients, mean CMT was 332.8 ± 173.9 *μ*m, 297.5 ± 166.3 *μ*m, 294.9 ± 121.8 *μ*m, 230.9 ± 107.0 *μ*m, and 220.4 ± 105.4 *μ*m at T0, T1, T2, T3, and T4, respectively. The mean CMT decreased significantly from T0 to T1 (*p*=0.0180), but no further regression was observed between T1 and T2 (*p*=0.8786). One month after switching, the mean CMT decreased significantly from T2 to T3 (*p* < 0.0001) and kept stable during the follow-up (T2 to T4, *p* < 0.0001) (see Figure 2).

For those switched to IVR, the mean CMT decreased from 297.9 ± 123.2 *μ*m at T2 to 240.0 ± 105.7 *μ*m at T3 and continued to decrease to 224.7 ± 107.0 *μ*m at T4. For those switched to IVB, the mean CMT decreased from 251.1 ± 122.0 *μ*m at T2 to 208.7 ± 106.7 *μ*m at T3 and 210.7 ± 100.2 *μ*m at T4. The mean decrease of CMT in IVR and IVB subgroup was 57.9 *μ*m and 42.4 *μ*m from T2 to T3 and 73.2 *μ*m and 40.4 *μ*m from T2 to T4. No significant difference was found between IVR and IVB subgroups for the mean decrease of CMT from T2 to T3 (*p*=0.53) and T4 (*p*=0.23) (see Figure 2).

**Figure 3:**
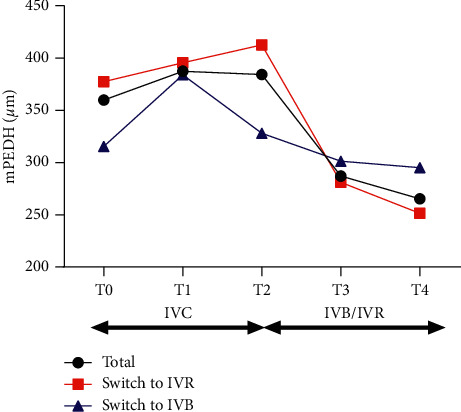
Changes of the mean mPEDH in eyes with persistent nAMD. The mPEDH did not change statistically significantly during the treatment of IVC. But it significantly reduced after switching to IVR or IVB from 384.3 ± 340.3 *μ*m to 287.2 ± 245.2 *μ*m at T3 and 265.3 ± 226.4 *μ*m at T4. The mean change of mPEDH from T2 to T4 was significantly larger in the IVR subgroup than that in the IVB subgroup (*p*=0.023). mPEDH, maximum PED height; PED, pigmented epithelial detachment; IVC, intravitreal injections of conbercept; IVB, intravitreal injection of bevacizumab; IVR, intravitreal injection of ranibizumab; T0, baseline visit; T1, 1 month after the first ICV; T2, 1 month after 3 monthly IVC; T3, 1 month after the first IVR or IVB; T4, 1 month after the last IVR or IVB.

For all the patients, there were no significant changes in mPEDH during the treatment of IVC (*p*=0.3482, 0.4520 between T0 and T1; T0 and T2, respectively). After switching, PED decrease from 384.3 ± 340.3 *μ*m at T2 to 287.2 ± 245.2 *μ*m at T3 (*p*=0.0018), 265.3 ± 226.4 *μ*m at T4 (*p*=0.0005). There was a significant reduction of mPEDH at the first month after switching and kept stable during the follow-up (Figure 3).

**Figure 4:**
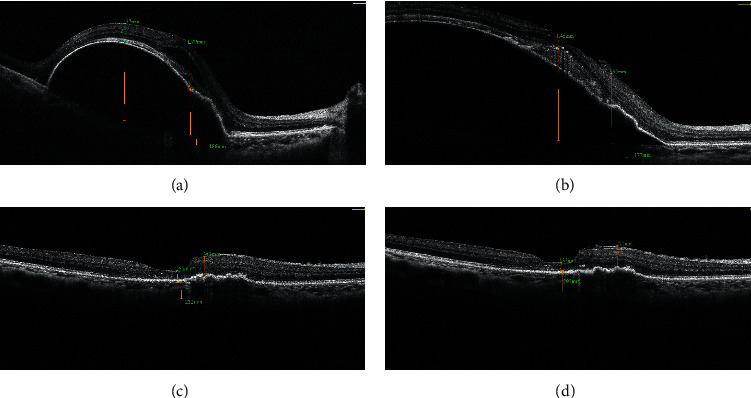
A representative case showed remarkable PED reduction after switch from conbercept to ranibizumab. (a) OCT at baseline visit showed large PED with SRF. (b) 1 month after the third IVC, PED did not improve but progressed; SRF became hyperreflective on OCT. (c) Patient was then switched to IVR. One month after the first IVR, PED dramatically decreased with SRF partially resolved. (d) After 3 monthly injections of ranibizumab, no SRF was present on OCT and only a very shallow PED was left. The total follow-up was eight months.

For those switched to IVR, the mean mPEDH decreased from 412.5 ± 398.3 *μ*m at T2 to 281.1 ± 225.8 *μ*m at T3 and 251.4 ± 201.7 *μ*m at T4. For those switched to IVB, the mean mPEDH decreased from 328.0 ± 275.7 *μ*m at T2 to 301.2 ± 289.6 *μ*m at T3 and 295.1 ± 281.8 *μ*m at T4. The mean changes of mPEDH in IVR and IVB subgroups were −131.5 *μ*m and −26.8 *μ*m from T2 to T3 and −161.2 *μ*m and −32.9 *μ*m from T2 to T4. The mean change of mPEDH of IVR subgroup from T2 to T3 was larger than that of IVB subgroup, but not statistically significant (*p*=0.058). Until the last visit at T4, the mean change of mPEDH from T2 was significantly larger in the IVR subgroup than in IVB subgroup (*p*=0.023) (see Figure 3). A representative case that showed remarkable PED reduction was shown in Figure 4.

**Figure 5:**
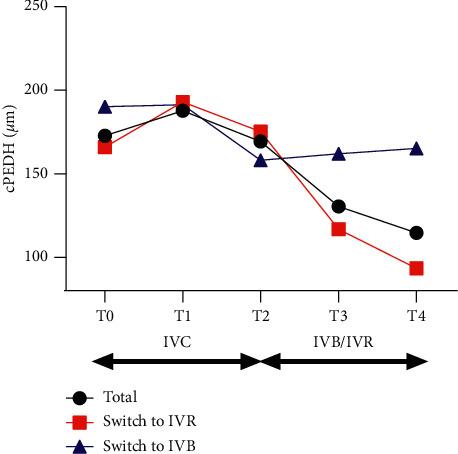
Changes of the mean cPEDH in eyes with persistent nAMD. cPEDH did not change statistically significantly during the treatment of IVC. But it significantly reduced after switching to IVR or IVB from 169.3 ± 230.6 *μ*m to 130.5 ± 180.2 *μ*m at T3 and 114.6 ± 142.6 *μ*m at T4. The mean change of cPEDH from T2 was significantly larger in IVR subgroup than in IVB subgroup at both T3 (*p*=0.033) and T4 (*p*=0.010). cPEDH, PED height at foveal center; PED, pigmented epithelial detachment; IVC, intravitreal injections of conbercept; IVB, intravitreal injection of bevacizumab; IVR, intravitreal injection of ranibizumab; T0, baseline visit; T1, 1 month after the first ICV; T2, 1 month after 3 monthly IVC; T3, 1 month after the first IVR or IVB; T4, 1 month after the last IVR or IVB.

For all the patients, mean cPEDH was 172.8 ± 244.8 *μ*m at T0, 187.8 ± 230.4 *μ*m at T1, 169.3 ± 230.6 *μ*m at T2, 130.5 ± 180.2 *μ*m at T3, and 114.6 ± 142.6 *μ*m at T4. There was no significant change in mean cPEDH during the treatment of IVC (T0 to T1, *p*=0.4187; T0 to T2, *p*=0.8624). After switching, the mean cPEDH showed a regression over time. The change of mean cPEDH was statistically significant between T2 and T3 (*p*=0.0227) and T2 and T4 (*p*=0.0097) (see Figure 5).

For those switched to IVR, the mean cPEDH decreased from 175.2 ± 259.6 *μ*m at T2 to 116.8 ± 191.1 *μ*m at T3 and 93.5 ± 136.0 *μ*m at T4. For those switched to IVB, the mean cPEDH decreased from 158.1 ± 127.6 *μ*m at T2 to 161.9 ± 146.1 *μ*m at T3 and 165.2 ± 148.1 *μ*m at T4. The mean change of cPEDH in IVR and IVB subgroups was −58.4 *μ*m and 3.8 *μ*m from T2 to T3 and −81.7 *μ*m and 7.1 *μ*m from T2 to T4. The mean change of cPEDH from T2 was significantly larger in IVR subgroup than in IVB subgroup both at T3 (*p*=0.033) and T4 (*p*=0.010) (see Figure 5).

**Figure 6:**
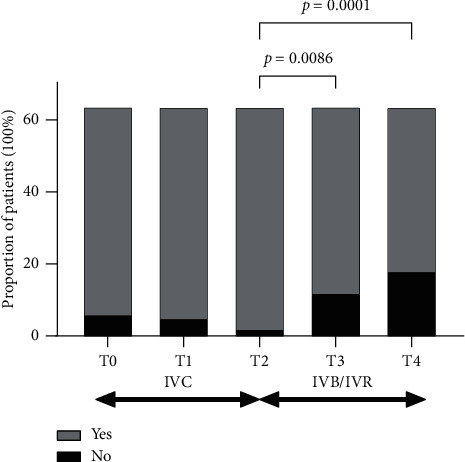
Changes in proportion of patients have IRF on SD-OCT. The proportion of patients with IRF did not change statistically significantly during the treatment of IVC. After switching to IVR or IVB, this proportion decreased significantly from 96.9% to 81.3% at T3 and 71.9% at T4. IRF, intraretinal fluid; IVC, intravitreal injections of conbercept; IVB, intravitreal injection of bevacizumab; IVR, intravitreal injection of ranibizumab; T0, baseline visit; T1, 1 month after the first ICV; T2, 1 month after 3 monthly IVC; T3, 1 month after the first IVR or IVB; T4, 1 month after the last IVR or IVB.).

For all the patients, the proportion of patients with IRF was slightly increased but not statistically significant before switching (90.6% at T0, 92.2% at T1, and 96.9% at T2). After switching, this proportion decreased significantly to 81.3% at T3 (*p*=0.0086) and 71.9% (*p*=0.0001) at T4 (see Figure 6).

**Figure 7:**
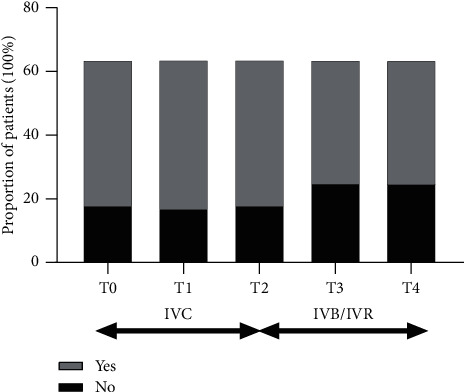
Changes in proportion of patients have SRF on SD-OCT. The proportion of patients with SRF did not change significantly before and after switching. SRF, subretinal fluid; IVC, intravitreal injections of conbercept; IVB, intravitreal injection of bevacizumab; IVR, intravitreal injection of ranibizumab; T0, baseline visit; T1, 1 month after the first ICV; T2, 1 month after 3 monthly IVC; T3, 1 month after the first IVR or IVB; T4, 1 month after the last IVR or IVB.

For all the patients, the proportion of patients with SRF did not change before switching (71.9% at T0, 73.4% at T1, 71.9% at T2). After switching, this proportion decreased slightly but not statistically significant (60.9% at T3 and 60.9% at T4, respectively) (see Figure 7).

Another subgroup analysis was made to investigate whether the type of AMD can affect the response to switching strategy. Eyes were divided into improved group and unimproved group according to the change of CMT, IRF, SRF, and mean BCVA at T3 comparing to T2. Improvement was defined as more than 10% decrease for CMT and more than 0.01 LogMAR increase for BCVA. Change of IRF or SRF was qualitatively graded by assessment of their amount on each OCT raster scan. Type 1, type 2, and mixed CNV groups were combined together in the statistic processing to avoid more than 25% of cells which have expected count less than 5. The proportions of improved response stratified by CNV type at 1 month after switching are listed in Table 2. No significant difference of response after switching was found between PCV and other types of CNV in our study.

No significant adverse event such as endophthalmitis, retinal detachment, induced cataract, or other systemic side effect such as increase in blood pressure and arterial thromboembolic events during the whole follow-up period (either with IVC, IVR, or IVB).

## 4. Discussion

Repeated intravitreal injection of anti-VEGF in the long term has been a major concern for the treatment of nAMD. This may be because of the persistent activity of CNV due to tachyphylaxis or tolerance to the medication. Tachyphylaxis is the lack of response when a drug is used repeatedly in a short period. When treatment is suspended for a short time, the efficacy of the drug can be regained but no response can be achieved by increasing drug dose [31–34]. On the contrary, tolerance is a progressive loss of drug efficacy after long-term use. The efficacy of a drug may be improved when the dose is increased or is administered at shorter intervals while the suspension of the treatment does not improve the efficacy. Development of tachyphylaxis in eyes receiving repeated anti-VEGF injections has been reported in several studies but very little has been reported about the prevalence of tachyphylaxis or tolerance. One study reported a 10% rate of tachyphylaxis in 58 patients treated with bevacizumab [32] and another study reported an incidence of 2% in 976 patients treated for 3 years with ranibizumab for AMD [33].

Eyes with persistent nAMD can benefit after switching from one anti-VEGF drug to another. Since ranibizumab and bevacizumab were available in the market earlier, most of the switching studies report outcomes for patients switching from ranibizumab and/or bevacizumab to aflibercept. Generally, they have shown anatomical improvement with stabilization of visual acuity. There were only data of few studies reporting “switching back” strategy [35–38]. SAFARI study demonstrated that switching from aflibercept to ranibizumab can also lead to a significant improvement of CMT with stabled or improved of visual acuity to nAMD patients with inadequate response [38]. But there has not been any relevant research about switching from conbercept to ranibizumab or bevacizumab. Our study is the first study that describes the functional and morphological outcomes of IVB or IVR after previous IVC for persistent nAMD patients. It showed reduction of CMT and PED and improvement of visual acuity after switching from conbercept to ranibizumab or bevacizumab for the persistent nAMD.

The following potential explanations for switching may lead to anatomical and/or functional improvement: first, the difference in molecular weight may result in different retinal penetration and systemic half-lives. The molecular weight is 143 kDa, 149 kDa, and 48 kDa for conbercept, bevacizumab, and ranibizumab, respectively [39]. In our study, IVR subgroup showed better response on mPEDH and cPEDH after switching than IVB subgroup. These results may be explained by the theory that smaller molecule is able to penetrate deeper and act more quickly than others. But in our study, there are also some eyes that showed response to bevacizumab after unsatisfactory response to conbercept, although bevacizumab and conbercept have similar molecular weight. This implies that the difference in molecular weight cannot fully explain the benefit after switching. Second, the difference in molecular structure may result in different binding affinities. Conbercept contains an extracellular domain 4 of VEGF receptors 2 which can improve the three-dimensional structure, increase the dimerization efficiently, degrade the isoelectric point, and prolong the clearance time of conbercept in the vitreous. Conbercept theoretically presents over 30 times binding affinity to VEGF compared to that of ranibizumab [40]. However, in our study, some eyes showed response to ranibizumab after unsatisfactory response to conbercept. This result implied that the benefit after switching may be attributable to other factors rather than different medicine, such as accumulative time effect. Third, genetic variations in the VEGF-A gene may cause inherent resistance to specific anti-VEGF agents. Genetic test was not evaluated in our study but theoretically it could partially explain our result. Fourth, tachyphylaxis to previous drug due to the development of neutralization of anti-VEGF may be stopped by changing to an immunologically different molecule [41].

The limitation of our study includes its retrospective uncontrolled design, small sample size, and short-term follow-up. Prospective controlled studies with larger number of cases and a longer follow-up are needed to optimize treatment decisions and the preestablished criteria are needed for a better understanding of switching to help us improve our therapeutic strategies.

In conclusion, our results indicate that treatment with bevacizumab or ranibizumab after previous conbercept regimen can lead to significant decrease in CMT, PED, and IRF and slight improvement of BCVA in a short period of time for persistent nAMD patients.

## Figures and Tables

**Table 1:** Patient demographic and baseline characteristics.

Characteristic	Number
Eyes (n)	64
Mean age (range)	70.3 ± 8.5 (53–92)
Women, *n* (%)	27 (42.1%)
Right eye, *n* (%)	34 (53.1%)

Type of AMD	

Type 1 CNV, *n* (%)	21 (32.8%)
Type 2 CNV, *n* (%)	5 (7.8%)
PCV, *n* (%)	25 (39.1%)
Mixed CNV, *n* (%)	13 (20.3%)

CNV, choroidal neovascularization; PCV, polypoidal choroidal vasculopathy.

**Table 2:** The proportions of improved response stratified by CNV type at 1 month after switching.

	Type 1 CNV	Type 2 CNV	PCV	Mixed CNV	*p* value
CMT	*n* = 21	*n* = 5	*n* = 25	*n* = 13	
Improved, *n* (%)	9 (42.9%)	3 (60.0%)	16 (64.0%)	8 (61.5%)	0.439
Unimproved, *n* (%)	12 (57.1%)	2 (40.0%)	9 (36.0%)	5 (38.5%)	

mPEDH	*n* = 18	*n* = 4	*n* = 24	*n* = 13	
Improved, *n* (%)	13 (72.2%)	3 (75.0%)	17 (70.8%)	7 (53.8%)	0.780
Unimproved, *n* (%)	5 (27.8%)	1 (25.0%)	7 (29.2%)	6 (46.2%)	

cPEDH	*n* = 10	*n* = 3	*n* = 19	*n* = 7	
Improved, *n* (%)	6 (60.0%)	1 (33.3%)	11 (57.9%)	3 (42.9%)	0.751
Unimproved, *n* (%)	4 (40.0%)	2 (66.7%)	8 (42.1%)	4 (57.1%)	

IRF	*n* = 21	*n* = 5	*n* = 25	*n* = 13	
Improved, *n* (%)	1 (4.8%)	1 (20%)	5 (20%)	1 (7.7%)	0.245
Unimproved, *n* (%)	20 (95.2%)	4 (80%)	20 (80%)	12 (92.3%)	

SRF	*n* = 21	*n* = 5	*n* = 25	*n* = 13	
Improved, *n* (%)	3 (14.3%)	0 (0%)	4 (16%)	1 (7.7%)	0.701
Unimproved, *n* (%)	18 (85.7%)	5 (100%)	21 (84%)	12 (92.3%)	

BCVA	*n* = 21	*n* = 5	*n* = 25	*n* = 13	
Improved, *n* (%)	9 (42.9%)	0 (0%)	12 (48%)	6 (46.2%)	0.604
Unimproved, *n* (%)	12 (57.1%)	5 (100%)	13 (52%)	7 (53.8%)	

CMT, central macular thickness; mPEDH, maximum PED height; cPEDH, PED height at foveal center; PED, pigmented epithelial detachment; IRF, intraretinal fluid; SRF, subretinal fluid; BCVA, best-corrected visual acuity; CNV, choroidal neovascularization; PCV, polypoidal choroidal vasculopathy.

## Data Availability

The data used to support the findings of this study are available from the corresponding author upon request.
